# Digital Futures in an Ageing Society: Frontline Perspectives on Sociotechnical Imaginaries in Swedish Eldercare

**DOI:** 10.1111/1467-9566.70159

**Published:** 2026-02-23

**Authors:** Freja Morris, Fredrika Thelandersson, Helena Sandberg

**Affiliations:** ^1^ Department of Communication Lund University Lund Sweden

**Keywords:** ageing population, care work, digital health, digitalisation, healthcare technology, promissory discourse, sociotechnical imaginaries

## Abstract

This article investigates how care professionals working with older adults in Sweden encounter, reproduce, and challenge sociotechnical imaginaries of a digitalised health and social care system. Drawing on interviews with 20 care professionals, we explore how promissory discourses that frame digitalisation as a solution to demographic and economic crises are taken up at the frontline of care. Despite regular experiences of technological malfunction and implementation challenges, care professionals maintain a persistent techno‐optimism and techno‐determinism in their narratives. We introduce the analytical concepts *tech love goggles* and the magic leap to explain how faith in digital futures is preserved amid present digital shortcomings and failures. *Tech love goggles* describes a tendency to idealise technology and deflect blame for its failures onto external factors, whereas *the magic leap* captures a future‐oriented logic in which present obstacles are expected to dissolve over time. Our findings highlight the performative power of sociotechnical imaginaries and suggest that optimism towards digital technologies can obscure the immediate needs and constraints of both workers and older care recipients. We argue for greater attention to the ethical and practical implications of deferring care solutions to an idealised digital future, especially for those whose time horizons are limited by age.

## 1 Introduction

This article examines how sociotechnical imaginaries of a digitalised health and social care sector are articulated, appropriated and contested by care professionals working with older adults in Sweden. Our findings show that these imaginaries are deeply rooted in the promissory narratives that dominate in public health policy discourse (cf. Petersen 2018b; Whitfield and Hamblin 2025; Lupton 2014). However, care professionals' narratives are not mere replicas of these discourses. Instead, they present as sociotechnical imaginaries that are actively reshaped in response to the practical challenges, ethical dilemmas and relational complexities that professionals encounter in their everyday work. Guiding our exploration of this theme are the following research questions: *How are sociotechnical imaginaries of a digitalised health and social care system sustained*, *stabilised and contested in local frontline care delivery? What are the implications of these imaginaries for care work*, *particularly in the context of ageing?*

The processes of ageing and digitalisation exist in health and social care policy discourse as a preordained pair. Within this discourse, the former poses as a problem—at the level of the individual as the deterioration of one's physical and cognitive abilities, and at the societal level as an impending demographic crisis that could potentially cause health and social care systems to collapse. Meanwhile, digitalisation at all levels provides a solution to these proposed problems. These promissory discourses (Petersen 2018a, 2018b) are both techno‐deterministic, in the sense that digital technologies are portrayed as inevitable paths to progress, and techno‐optimistic, in the sense that digital technologies are considered inherently beneficial and capable of solving complex and multifaceted societal challenges. Within this framework, technology is assumed to enable older people to live more independently, improve care quality and ease pressures on health and social care personnel.

This optimistic framing has been critically examined by scholars who argue that such narratives obscure the complexities of health and social care systems, downplay organisational and infrastructural constraints and understate the diverse needs and capabilities of older adults (cf. Eccles 2021; Heidlund and Sundberg 2023; Neven and Peine 2017; Mort et al. 2013; Valokivi et al. 2021). Research studies have demonstrated that implementation, far from being seamless, is often marked by delays, unintended consequences and outright failure (Gibson et al. 2019; Pols and Willems 2011; Lupton 2014). Nonetheless, the rhetoric surrounding health and social care digitalisation remains aspirational and future oriented.

Clearly, implementing digital health technologies is more complicated and prone to failure than what techno‐optimist visions purport. Interestingly, this does not necessarily quell techno‐optimism among the actors who experience this dissonance. In our interviews with 20 Swedish care professionals working with older care recipients, we found that techno‐optimism and techno‐determinism prevailed in the interviewees' narratives.

This article thus sets out to analyse the specific local forms that techno‐optimist and techno‐determinist narratives take among these actors who frequently experience failure, maladaptation and setbacks in their interaction with digital technologies. These actors are at the receiving end of both the fulfilled and unfulfilled promises that these technologies offer. Our findings suggest that sociotechnical imaginaries of a future smooth‐running and efficient digitalised health and social care system enter into their accounts of their work and even informs how they understand technological failures and misapplications. We propose two analytical concepts to explain this persistence of optimism for a digital future in the face of a sometimes‐erratic digital present: *tech love goggles* and *the magic leap*. The former describes how present‐time failure of digital technology is almost never ascribed to faults in the technology itself, but in circumstances, context and users. The latter captures the temporal logic by which current challenges are portrayed as simultaneously insolvable in the present and destined to be solved in the future.

Sweden provides a particularly germane case to study as it has profiled itself as world‐leading in health and social care digitalisation. In 2017, Sweden launched a national strategy, *Vision for eHealth 2025*, with the stated aim that ‘in 2025, Sweden will be best in the world at using the opportunities offered by digitalisation and eHealth’ (Vision eHälsa 2025). The ‘vision’ presented is indeed desirable: facilitating individuals' participation in their own care, simplifying communication between care providers and recipients, and increasing organisational efficiency (Vision eHälsa 2025). To what degree this ‘vision’ has been realised is not our concern here, but suffice to point out that significant government efforts and resources have already been invested in digitalising Swedish health and social care (cf. Arnelid 2025). The Swedish health and social care system is mainly publicly financed, universal and decentralised. The implementation of digitalisation is largely delegated to regional and municipal authorities, which places frontline staff at a key juncture between national and local strategy and practical delivery.

## 2 Theoretical Framework: Technology, Futurity, Imaginaries

From the printing press to artificial intelligence, narratives of technological innovation routinely conjure up notions of inevitable progress. In these techno‐deterministic narratives, technological change is seen as an ‘independent factor, impacting on society from outside society’ (MacKenzie and Wajcman 1999, 5). Technological progress, then, is often seen as concomitant with, and determining of, human progress.

The linear, progressivist and deterministic assumptions underlying these conceptions of socio‐technical change have been critically examined by social scientists, particularly those working within the sociology of the future and sociology of expectations (Brown et al. 2000; Borup et al. 2006; van Lente 2012; Tutton 2017). Informed by Science and Technology Studies (STS), these scholars argue that the practices of predicting, imagining and visualising the future are not neutral or purely speculative exercises, but are performative acts that shape the present and the future by structuring and influencing decisions‐making, investment and governance. Additionally, that some technological futures are cast as inevitable means that other futures are kerbed, ignored or delegitimised (cf. Schiølin 2020; Vicsek 2021; Brown et al. 2000). Ultimately, these scholars argue, how we visualise the future in the present affects what kinds of futures are possible.

### 2.1 Sociotechnical Imaginaries

One of the most influential conceptual understandings of these futurescapes and their ordering effect in the present was developed by Jasanoff and Kim in 2009 and then elaborated upon by the same authors in 2015: the full definition of *sociotechnical imaginaries* is that they are ‘collectively held, institutionally stabilised, and publicly performed visions of desirable futures, animated by shared understandings of forms of social life and social order attainable through, and supportive of, advances in science and technology’ (Jasanoff 2015, 4). The concept thus seeks to amalgamate notions of imagination and discourse, that traditionally focus on cognitive processes and language respectively, with a sensitivity to how material artefacts, flow of resources and institutional practices participate in the co‐production of these shared visions of desirable futures.

Recent scholarship has highlighted sociotechnical imaginaries as multiple and contested *as well as* collective and shared (cf. Mager and Katzenbach 2021; Sismondo 2020). Always locally and materially embedded, visions of sociotechnical imaginaries emerge in a myriad of ways (Jasanoff 2015). An emerging body of literature has investigated sociotechnical imaginaries in healthcare, where the stakes of technological futures are deeply tied to questions of ethics, governance and human life. Sociotechnical imaginaries have been employed as a lens for examining such things as AI (cf. Hoff 2023; Watson and Wozniak‐O’Connor 2025), big data (cf. Gardner 2023; Stevens et al. 2022) and robotics (cf. Arnelid 2025; Breuer and Müller 2024). When sociotechnical imaginaries of healthcare technologies are explored in policy, industry and media discourse, studies show that there is a strong tendency towards optimistic and deterministic framings of healthcare technologies as solutions to healthcare systems in crisis. These studies tend to emphasise how sociotechnical imaginaries have performative effects through how they legitimise reforms and investments that affect the very ways in which we not only conceive of, but also structure, fund and carry out health and social care (Breuer and Müller 2024; Hoff 2023; Watson and Wozniak‐O’Connor 2025).

Other studies explore how these sociotechnical imaginaries are taken up in clinical and organisational settings. Here, the local and multifaceted specificities of healthcare technology implementation often challenge the sweeping, deterministic and simplistic imaginaries that accompany them. Mwale and Farsides (2020) found that British general practitioners struggled to identify with the strongly promissory narratives of genomic medicine, creating a gap between ‘work as imagined’ and ‘work as done’. Schwennesen (2019) argued that algorithmic imaginaries must be continuously enacted and repaired through situated practices in clinical encounters. Gardner (2023) and Stevens et al. (2022) both highlight the affective dimensions of sociotechnical imaginaries for professionals. The former makes use of the notion of enchantment to explain how hospital staff remain invested in the idea of a data‐driven future, even amid infrastructural breakdowns and tensions. The latter, meanwhile, explore how the metaphor of ‘dreams’ can help us shed light on how professionals affectively engage with the grand promises of data‐driven healthcare. They identify four recurring ‘data dreams’—of being seen, timeliness, connectedness and control—which reveal how professionals internalise, reinterpret and sometimes challenge the optimistic discourses surrounding big data in healthcare.

### 2.2 Applying Sociotechnical Imaginaries

Sociotechnical imaginaries as a concept offers some specific entryways to our study: Firstly, it draws on performative understandings of futurity, which allows us to consider the implications of visions of the future for a digital health and social care *in the present*. Secondly, by focussing on *desirable* futures, it highlights the techno‐optimism that tends to permeate imaginaries of technoscientific innovation (albeit with its dystopian double chronically lurking in the shadows) and that stands out in our interviewees' narratives. Thirdly, by emphasising that sociotechnical imaginaries are simultaneously multiple and institutionally stabilised, we are afforded an opportunity to explore how far‐reaching, shared, and stabilised visions materialise in specific forms in local contexts.

Methodologically, Jasanoff (2015) suggests we mine diverse prominent and influential genres such as policy documents, mass media, popular writing, and more in order to grasp the outlines of existing sociotechnical imaginaries. Accordingly, previous research studies on discourses, narratives, sociotechnical imaginaries and myths surrounding digitalisation in healthcare will serve as point of departure to understand how these visions are locally adopted and enacted among care professionals.

## 3 Care, Technology, and Discourse

### 3.1 The Promissory Discourses on Health, Care and Technology

Concerns about rising costs of health and social care, ageing populations and workforce shortages span across most countries in the Global North. In response, policy narratives across Europe and beyond increasingly promote digital technologies as central solutions to systemic challenges. These discourses frame digitalisation as inherently future‐oriented and progressive, with digital health technologies positioned as the key to more efficient, cost‐effective and patient‐centred care (cf. Whitfield and Hamblin 2025; Lupton 2014; Greubel et al. 2021). Despite national differences, policy documents, media portrayals and innovation strategies from diverse contexts reveal strikingly similar framings of digital health. Researchers have identified a dominant narrative of a *crisis* in health and social care and an *optimism* as well as a *determinism* concerning technology's role in providing a solution (Petersen 2018b; Whitfield and Hamblin 2025, for a discussion of national differences within this broad optimism and determinism, see Valokivi et al. 2021). Although the specific technologies vary—from telecare and eHealth to welfare technology—their associated promises remain consistent: increased efficiency, cost savings, improved quality of care and enhanced independence and empowerment for care recipients (Eccles 2021; Lynch et al. 2019; Mort et al. 2013; Kamp et al. 2019).

Studies have shown that these promissory discourses particularly dominate public and policy narratives in relation to the ageing population (cf. Whitfield and Hamblin 2025; Valokivi et al. 2021; Greubel et al. 2021). Neven and Peine (2017) have referred to the discursive framing of a demographic crisis and technological solutionism as *the ageing‐and‐innovation discourse* and emphasise that this discourse is performative as well as descriptive. It constructs a moral and economic ‘triple‐win’: society benefits through reduced care costs, older adults gain independence and better care, and the economy thrives through technological growth. However, the same discourse carries what the authors call a ‘triple sin’: it treats all technologies as inherently beneficial, frames ageing in predominantly negative terms and embeds moral imperatives that make critique difficult.

### 3.2 Care Professionals and Digital Health Discourses

The professionals who work with implementing digital technologies in care work are placed at the intersection of institutional discursive promises and the local, multifaceted and materially embedded practices of care work. Research studies show that when new technologies are introduced, they rarely perform as predictably as institutional narratives suggest, sometimes leading to a ‘mismatch’ between institutional promises and their ‘material realisations’ (Roberts and Mort 2009). Pols and Willems (2011), described how a new telecare tool behaved quite differently in practice—it had to be domesticated and ‘tinkered’ with to fit local routines, and it also unleased unforeseen effects on care delivery. Likewise, Mort et al. (2013) find that ‘good care’ through telecare only works through intensive shared work by staff, users, and devices, making implementation a highly situated practice rather than a one‐size‐fits‐all solution (see also Gibson et al., 2019, who have referred to the idiosyncratic ways that family carers utilise assisstive technologies as ‘bricolage’).

Despite on‐site tinkering practices, professionals must navigate the powerful discursive currents that portray digitalisation as inevitable and unequivocally positive. As with professionals' engagements with sociotechnical imaginaries discussed earlier, research shows that professionals engage with promissory discourses in different ways and are not passive recipients of these narratives. Hamblin et al. (2025) analyse tech‐solutionist discourses at three levels in the UK (policy, sector stakeholders, and homecare managers and care workers) and find that the optimist narratives prevail at the macro‐ and meso‐levels, emphasising technology's capacity to enhance efficiency and quality. At the micro‐level, however, ambivalence enters as care workers describe new devices that generate extra work or hidden responsibilities, undercutting the efficiency gains promised by policy. Højlund and Villadsen (2020) document that eldercare workers employ a range of strategies—from resistance to non‐use to enthusiastic adaptation—in order to integrate new devices on their own terms. Crucially, they insist on preserving core care ethics, even when lifts or smart toilet. alter routines, care staff focus on person‐to‐person interaction and ‘proximity’ care as non‐negotiable. Lynch et al. (2019) show that practitioners acknowledge the policy valorisation of independent living, but note that older users define independence very differently and they caution that the simplified narrative can conflate policy aims (e.g., cost‐saving vs. user empowerment), sometimes undermining clients' needs.

### 3.3 Contextualising the Problem: Sweden as a Case

The promissory discourses and sociotechnical imaginaries that dominate Swedish policy documents have been extensively researched, both at the regional level and at the national level (cf. Tucker 2023; Valokivi et al. 2021; Nilsson 2023; Kamp et al. 2019; Arnelid 2025; Nilsson et al. 2024; Frennert 2020; Cozza et al. 2019). These studies show that Swedish digital health discourses echo their international counterparts: they are saturated with deterministic and optimistic assumptions, portraying technological progress as both inevitable and desirable.

Swedish scholars have carefully studied the tensions and contradictions embedded within these promissory discourses. Frennert (2020) and Arnelid (2025) both argue that welfare technologies—although framed within similar solutionist imaginaries—are in practice received and imagined differently by different actors, in part influenced by institutional factors, such as fragmented care organisation, funding limitations and implementation strategies. Cozza (2024) analyses how datafication of eldercare contributes to the performance of the ‘care crisis’ by framing care through metrics and algorithms that follow managerial logics rather than relational logics, affecting the meaning and experience of care. Nilsson et al. (2024) also highlight the preformative power of what they refer to as the ‘digital technology solution discourse’ in Swedish health and social care policies. By framing digital technology as the inevitable and desirable solution to demographic and resource challenges, these discourses shape policy priorities, allocate responsibility to individuals and marginalise alternative approaches to care (Nilsson et al., 2024).

## 4 Methods

The data that our arguments are based upon derives from twenty semi‐structured interviews with different local care professionals working with older citizens (see Table 1). Our sample consisted of different care professionals whose work with older adults is in different ways mediated by digital technologies. The majority of our participants were women (*N* = 18) and the ages ranged between 23 and 69, with an average age of 48. Although the vast majority of our participants worked in the municipal social care sector, a couple of specialist nurses worked in the regional healthcare sectors but these were recruited because they belonged to a national association for eldercare and contributed with a more overarching perspective on eldercare and digital technologies.

**TABLE 1:** Participant overview.

Occupation	*N* (20)	Place of work
Care manager	2	Municipality
Specialist nurse (dementia) and nurse	2	Municipal home care services
Auxiliary nurse	8	Municipal home care services
Physical and occupational therapists	3	Municipal home care services
Specialist nurse (geriatric and dementia)	5	Municipal and regional care services

The interviews were conducted over the course of 6 months, February–November 2024. Most participants were interviewed in‐person on site at the workplace of the participant (*N* = 14) and some were interviewed through video conferencing software or telephone (*N* = 6) in order to overcome considerable geographical distance between participant and interviewer. All interviews were audio recorded and later transcribed verbatim by a professional communications company that adheres to strict ethical standards and is approved for handling sensitive research data. Written informed consent was collected before the interviews. The project has been approved by the Swedish Ethical Review Authority (no 2022‐05780‐01). NVivo was used for coding and analysis of the transcribed and anonymised interviews.

The interviews were conducted as part of a larger Nordic research project. The aim of these particular interviews was to explore how care professionals, in their work with older care recipients, make use of digital technologies, and what opportunities and challenges they face in providing equal health for all. The same interview guide was used for all interviews and contained open‐ended questions about such things as how the health professionals worked with digital technology, whether and how they help older adults with digital technologies, their perception of the opportunities and challenges of digitalisation for older citizens and their overall opinion of digitalisation in health and social care.

Our data were coded and analysed using an abductive approach (Tavory and Timmermans 2014), a method that aims to generate new theoretical insights by moving iteratively between theory and data in search of the surprizing or unexpected. During our initial phase of open coding, the broad and nebulous code ‘techno‐optimism’ caught us a little off‐guard amidst the many reservations and descriptions of inadequate technology. We were also surprized to find that there were no notable differences between the professional groups, their age categories, or other demographic factors in expressing these sentiments. We explored this theme in relation to a range of sociological theories, remaining theoretically ‘agnostic’, engaging with our data in an abductive, back‐and‐forth manner between data and theory (Tavory and Timmermans 2014). For instance, studies on health and social care technology discourses helped us unearth how these discourses were echoed by the care professionals. However, to understand the persistent future optimism in the face of present challenges, the patterns that we identified in our data were best developed and described with the help from the sociology of the future and expectations and particularly the concept of sociotechnical imaginaries, which served as a lens to develop our understanding of how ‘scientific and technological visions enter into the assemblages of materiality, meaning and morality that constitute robust forms of social life’ (Jasanoff 2015, 9). The concrete mechanisms through which optimism persisted in face of technological challenges and failures needed further conceptual tools, which led us to introduce *tech love goggles* and *magic leap* as conceptual tools to describe these mechanisms.

## 5 Findings

Across our interviews with care professionals, we identified a strong and persistent belief in digital technologies as an inevitable and necessary solution to the challenges facing the welfare state. All our participants expressed concern with not only the current state of Swedish care institutions but also the future conditions for care work. We found no significant differences between how the different groups of care professionals related to digital promises and futures of care work, nor any differences based on age or geographic location. Although our sample size leaves no room other than for speculation, we may reflect that the sweeping nature of techno‐optimist and techno‐determinist predictions as well as the far‐reaching problem formulation of a health and social care sector in crisis, gives rise to similar enactments of sociotechnical imaginaries across different demographic and professional variables. The quotes from our interview persons are accompanied by information about gender, profession and age in order to retain empirical closeness and showcase the demographic range of our participants.

Throughout their narratives, a sociotechnical imaginary often emerged of a frictionless digital future where Swedish care institutions operate like a smooth‐running machinery. Within this future seamless system, all significant actors align—patients, professionals, management, technology and infrastructure. Current challenges—such as decreasing manpower, quality of care, independence of older people, the economic state of the health and social care system—have been obviated through technology. In reality, of course, digital technologies are already deeply integrated into the daily work practices of our interview participants. Digital technologies are used for communicating with colleagues within and across the health and social care sector, for communicating with care recipients and their families, for storing and circulating information, for streamlining multiple care needs, for overcoming physical distance, for outsourcing specific care tasks (such as routine nightly check‐ins through video camera as opposed to physical check‐in), etc. Moreover, some of the current challenges that our participants described were technological in nature, for instance incompatible digital systems, subpar technological performance, or arduous interfaces. In our findings, current technological problems and technology's current inability to solve the structural problems of care work did not diminish our interview persons' hopes for *future* success for digitalised care.

In the first part of this section, we demonstrate how techno‐optimist and techno‐determinist narratives dominated our participants' framing of both problems and solutions within health and social care. In the second part of this section, we go more deeply into understanding how sociotechnical imaginaries of digitalisation and care were kept alive despite current tensions, issues and outright failures of digital technologies. Taking as our point of departure Jasanoff and Kim’s (2015) conception of sociotechnical imaginaries, we seek to demonstrate that care professionals' narratives do not simply reflect broader promissory discourses but rather partake in the co‐production of these imaginaries in ways that are specific to the socio‐material contexts that they work in. Specifically, we identify two narrative strategies—that we call *tech love goggles* and *the magic leap*—that we identified that our participants employed and that helped them maintain a future‐oriented optimism and determinism regarding technology's capacity to solve their current problems, in spite of frequently experiencing technological failure or friction in the present and the shortcomings of the current state of digitalisation.

### 5.1 A Seamless Future for a Chaotic Present

The scene that is set by the care professionals is one where the present is near‐catastrophic and the future is perilous. The way to avoid this dystopian future is framed in *techno‐determinist* and *techno‐optimist* terms. Techno‐determinism takes two shapes in our data: one is a positive enlightenment lens where technological innovation is emblematic of societal progression or ‘what drives us forward, no matter how you look at it’, as one auxiliary nurse expressed it. The other shape that technological determinism takes is through an austerity lens. Here, technology is cast as the only viable response to dire future prospects with regards to financial and human resources. In both these techno‐deterministic renderings, technology is framed as inevitable and as the only way forward. A specialist dementia nurse explained that digitalisation was urgent and imperative, which she motivate within a framework of structural crisis:

It [the impending crisis and imperative to digitalise] is being talked about all the time in healthcare. We're facing… I mean, in just five or six years, there will be a lot more people, and then we'll have a total crisis if we don't do something. But the solutions aren't within reach—there aren't the financial or time resources to even dare to try. So maybe it's about a closer collaboration between municipalities and universities or innovators, so there's actually a space to test things and say, ‘this works’

(Specialist dementia nurse, female, 46 years)

The health and social care sectors were consistently described as being in a state of perpetual crisis by our participants. Similar to the discourses identified in previous studies, our participants understood the causes of the crisis as a toxic blend of human and financial resource deficit as well as an ageing population whose medical conditions are becoming increasingly complex and demanding. This diagnosis of the current state was often invoked by our participants as a backdrop for explaining the necessity and purpose of digitalisation. In the conceptualisation of diagnosis (systemic crisis) and treatment (digital technologies), the latter frequently assumed the character of a ‘silver bullet’ solution. In an analysis of UK telecare policy documents, Eccles (2021) identified a silver‐bullet thinking, wherein the efficacy of technology is assumed rather than scrutinised in relation to the particular context of implementation.

[Digitalisation is] A must, as I see it. In part, we need to keep up with the times. We need to because we have an ageing population. We’re going to need… and there are fewer of us who will be working. Soon we’ll have no staff left. And in the future, we won’t have much staff at all. So we need to find smart solutions. We also need to integrate AI into health and social care much more. Unclear how, but it’s a must.

(Specialist nurse, female, 60 years)

We need to do much, much more. We are really facing a huge crisis. We don’t have health and social care staff, we don’t have solutions. We have to do something, and we have to do it as soon as possible. We need to look much more at digital home visits from home‐care services. For example, medication‐dispensing robots. Maybe we could rent out robot vacuum cleaners. We need to look at smart solutions. And I myself say that when I get old, I only want to be cared for by robots and AI. And then hopefully one of my children can place an order—if I can’t speak for myself—about how I want these robots to behave towards me. Because I don’t think we will have the human resources.

(Specialist nurse, female, 46 years)

In these quotes, techno‐determinism is strongly linked to techno‐optimism. The ‘smart’ solutions that are called for refer to technological advancements that are efficient, flexible, and scalable. Both nurses also place high hopes and expectations are placed on artificial intelligence. The confidence they hold for this incipient and new technology (at least as far as their work environment goes) is striking. Within the sociology of expectations, it has been shown that ‘high or optimistic expectations is discursively correlated with a technology in its infancy’ (Brown and Michael 2003, 12). AI is currently the technological flagship of innovation and digital transformation in nearly all areas of society, including health and social care. Previous research studies have demonstrated how the sociotechnological imaginaries concerning AI emphasise its inevitability and unprecedented novelty, creating a sense of urgency to act (Watson and Wozniak‐O’Connor 2025; Hoff 2023; Bareis and Katzenbach 2022). The specialist nurse cited above echoes this rhetoric: it’s a must to integrate AI into health and social care, even though the utility and implementation are left unstated and uncertain.

It is important to note techno‐optimism does not refer to a sentiment among our interview persons that technology is inherently good or always best practice. Rather, it refers to an optimism regarding technology's ability to solve specific problems, such as decreasing manpower or communication challenges.

### 5.2 Sustained Optimism in the Face of Current Challenges

Our participants already work in a highly digitalised work environment. In many ways, the digitalised future that they have been promised for many years is already upon them and in motion. However, the present state of digitalisation was not described as quite as smooth and friction‐free as the digitalised future yet to come. In fact, sometimes the problems they were having pertained directly to the digital technologies they utilised in the current state of digitalisation. Interestingly, despite frequent frustrations with malfunctioning or underperforming technologies, the care professionals we interviewed consistently expressed strong confidence in digitalisation as the inevitable and necessary future of care. Rather than losing faith when tools failed or created more work, participants deflected blame or deferred resolution. This paradox—persistent optimism in the face of technical failings—was a consistent feature across interviews. In what follows, we describe two narrative techniques that our participants employed in order to keep the promise of future digital relief intact in the face of an imperfect digital present.

#### 5.2.1 Tech Love Goggles

The concept of *tech love goggles* captures how digital technologies are viewed through a lens of optimism and inevitability even when they fail. The concept draws on the idiom ‘love goggles’, where one sees a person with whom one is infatuated through a glorifying and idealising filter, tending to ignore or downplay their flaws. In our material, this is most evident when the participants talked about failures or problems with technology. In these instances, failures were explained in terms of context, user limitations, or lack of organisational readiness. In the following quote, an auxiliary nurse recalls an incident of a technology that failed at providing the support it was meant to deliver:

Participant *We’ve tested quite a few different things,* like GPS alarms and all sorts. Well, we are the first point of contact with the care recipients, so maybe we’re the ones who could help develop this technology. *Because there’s a lot more that could be done, I believe.* We’ve tested GPS. [The care recipients] wore this kind of watch, in case they went outside and wandered off

I And how did it work, would you say?

P *Poorly, unfortunately.* Because we were out running in the forest, me and a colleague, thinking he had gone into a lake. But that wasn’t the case at all. He was sitting in his armchair at home watching TV, so there wasn’t an issue

(Auxiliary nurse, female, 42 years)

The GPS alarm, presented as one out of multiple technologies they had tested, did not give the care professionals reliable information about the whereabouts of their wearers. This technological failure also led to extra work for the auxiliary nurse and her colleague. Nonetheless, the auxiliary nurse remains convinced that ‘there is a lot more that could be done’. A little bit further on in the interview, she decides that the issue of the failing GPS alarms is a problem of poor procurement from the municipality's side, implying that there are effective GPS alarm systems that the municipality could have procured. The assumption that there were easy, reliable, and accessible technological solutions available out there but that these had just not been procured and implemented here and now, was a recurring idea in our data.

Then one can think that it’s a bit cumbersome to have so many apps… [laughter] when technology is advancing and one could do everything. But it would have been enough… just a fingerprint, and that would have solved a lot for us.

(Auxiliary nurse, female, 51 years)

Well, the communication is clearly lacking today. We have different medical record systems: the region has one, the municipality has one, we have an intermediate system where we can go in and read. I just don’t understand why we don’t have the same system. I mean, why aren’t we just one? There are just too many communication channels, I would say. It should be possible to make it simpler through digitalisation.

(Physiotherapist, female, 40 years)

In these quotes, technology is envisioned as offering the harmonisation of many different functions, making the work more smooth‐running and simpler. Many studies of sociotechnical imaginaries and discoursers surrounding digital health and social care identify a tendency to gloss over local variations and complexities (cf. Petersen 2018b). As we see in the quotes above, this leads to a simplified understanding not only of technology's integration in the health and social care system, but also of the technology itself. In both these quotes, digitalisation is implicitly understood as an easy solution and, well to note, one that is already available if only the right decisions are made within the organisation. Quotes like these frame the problem of technology as mainly *bureaucratic* problem, wherein the ‘right decisions’ regarding technology have not been made, rather than a problem of the technology itself.

In a different iteration, blame for technological failure was not only placed on organisational factors but also on the users of the technology.

The biggest obstacle is probably their own abilities. For example, if you start developing dementia or begin losing your vision, hearing, or sense of touch—yeah, then it immediately becomes much harder. So the tool itself isn’t really an obstacle, as long as you’re physically and mentally okay. It’s more about how you are as a person… I think it’s more about where you are in your ageing process. Yeah, and interest, of course, also plays a role. Then we can also play a big part in encouraging and supporting our care recipients to… But again, it still comes down to us getting the conditions and opportunities to help our care recipients. We need to be given that time.

(Auxiliary nurse, male, 50 years)

Similarly, when one nurse was asked what she thought were the biggest challenges or obstacles in relation to older adults and digital technologies, she replied:

I think that many times it’s fear, that something is unfamiliar. So they’re afraid it will be complicated and that it’s something new and so on. And then I also think that you can provide support by helping to install something and, well, just explain. Because many systems are very, very pedagogical.

(Specialist nurse, female, 53 years)

One of the grand promises of digitalisation in the health and care sector is that it will make interaction and communication with care recipients more efficient—video consultations, online booking systems, medicine robots, etc. are examples of technologies that hold great promise and that were to various degrees already implemented in the settings where our participants worked—with varying success. Seeing technology through tech love goggles, our participants sometimes understood the causes for the failures of such technologies not as a technical issue, but a human issue. The same humans, we might add, that the technologies in part are developed to help are framed as the impediment to the technologies' success.

The idealisation of technology through tech love goggles is a product of the broader sociotechnical imaginary, in which digital technologies are understood not just as tools, but as inherently good, progressive, and morally desirable. When technology is framed as self‐evidently valuable, it rarely needs to be justified or evaluated. The underlying logic is circular: if technology fails, it must be because something else got in the way. In this way, the inherent value of technology is protected from critique while responsibility for its failures is redistributed—onto the older users, their perceived lack of digital literacy, lack of resources or failed initiative in the health and social care system.

#### 5.2.2 The Magic Leap

The second mechanism underpinning sustained optimism is what we call *the magic leap*. It describes how something is portrayed as simultaneously insolvable in the present and destined to be solved in a digitalised future. It therefore relates both to techno‐determinism narratives—in that the digitalised future is conceived as inevitable—and techno‐optimist narratives—in that the digitalised future will be superior to the digitalised present. Within this narrative framework, current technological hurdles are conceived of as temporary glitches, implying that a fully digitalised eldercare will eventually run smoothly once society catches up with the technology or invests in the right kind of technology. The magic leap therefore has affinities with the silver‐bullet thinking that Eccles (2021) identified in British policy documents that was described above. Whereas the silver‐bullet thinking refers to an overly simplified causality relation between implementation of technology and its outcomes, the magic leap refers to a different kind of causal effect—one where time itself assumes the role of transformative agent. In the magic leap, if we just wait, some of the most pressing issues with the implementation of digital technologies will have resolved themselves:

Then it will probably change, of course, more and more, because you get used to it, so it will get better over the years.

(Occupational therapist, female, 57 years)

But I suppose it’s a transition, that those people have not reached that point yet… so that as the years go by or as time passes, people will get better, I reckon.

(Care manager, female 33 years)

It is noteworthy that the dissolution of the problematic will require no work or resources but will happen naturally and effortlessly. It would seem that there is a *magic leap* from a problematic present to a smooth future. All that is needed to get from problematic present to smooth future is *time*. Time is imagined as an autonomous force that will inevitably resolve the frictions of the present. This agentic capacity of time is familiar to sociologists of the future and expectations:

Just as commonly, agency is projected into the future itself. Examples of this kind are implied in the grammar of the future through which actors speculate upon ‘what the future will bring’ and near‐certain expectation of what the future ‘will be like’. Here time itself is reified into acting determinately raising broader questions of how it is that agency becomes a property of time and the ‘the future’ rather than the ordering practices that produce it. Such practices thus become concealed behind the future acting as a powerful agent on our present.

(Brown et al. 2000, 9)

Time as a precondition for the magic leap from the problematic to unproblematic rests on a somewhat morbid assumption: that this older generation will have died out and been replaced with a new more tech savvy generation of older people. We should also observe that within this line of thinking, it is the *older people* who get better in time, not the technology. Humans need to adapt to the technology rather than the other way around. In these quotes, it is assumed that humans will inevitably progress with technology. However, we are well to note that there is the implication of a time lapse between the technological development and the human progress, that is, the humans are a little bit slow in progressing in line with the technological advancements. The verdict is nonetheless that humans will eventually catch up, reinforcing a technologically deterministic understanding of the interaction between technology and society (cf. MacKenzie and Wajcman 1999).

Another assumption that underlies this view on human and technological advancement is that the development of digital technologies has somehow reached its peak and will remain at a plateau where people will have time to catch up. Paradoxically, this goes against the view of technology as an ever‐evolving catapult that throws us into the future. If technology was conceived as moving at the same speed as humans, then humans would never catch up because the next older generation would always be one step behind the latest technological advancement. Empirical studies have indeed shown that older adults use of ‘old technologies’, even digital one's, poses a problem for this groups' digital participation (cf. Søgaard Nielsen 2025).

## 6 Discussion

Our findings show that care professionals draw upon techno‐determinist and techno‐optimist narratives when conceiving the digital future of health and social care. Digital technologies are perceived as imperative to meet the future demands for manpower, efficiency and distribution of resources. We found these sociotechnical imaginaries of a seamlessly digitalised future particularly interesting seen in relation to the fraught and often complicated digital present that they worked in. Despite repeated encounters with technological shortcomings, our participants rarely questioned the core belief that digital technology holds the key to resolving the structural challenges of the health and social care system. Instead, they framed failures in ways that preserved the legitimacy of the technology and deferred fulfilment to a more perfect future.

In order to explore this paradox, we searched for instances where present‐time technological frictions or failures were discussed in order to conceptualise how techno‐optimism and techno‐determinism are maintained in the face of friction, failure, and broken promises. In our interviews, the promise of technology was preserved through a tendency to idealise technology and downplay or externalise its flaws (*tech love goggles*) and a conviction that unresolved issues today will naturally be solved in the future (*magic leap*). Together, these two modes of thinking allow professionals to uphold the belief that more technology is both unavoidable and ultimately beneficial, even when their experiences with current tools are problematic.

Previous research studies on sociotechnical imaginaries and promissory discourse in health and social care has predominantly examined policy documents, industry narratives, innovation strategies, and (to a lesser extent) managerial and organisational actors. This body of work has been essential for showing how particular technological futures are rendered desirable, inevitable and actionable. Far less attention has been paid to how these imaginaries are sustained and stabilised by frontline care professionals—despite longstanding recognition in medical sociology and STS that frontline workers do much of the work required to reconcile institutional visions with practical realities. Our findings therefore bridge scholarship on sociotechnical imaginaries with work on ‘care in practice’ and the mundane labours of tinkering, maintenance and repair (e.g., Mol 2008; Mol et al. 2015).

Specifically, we demonstrate how repair work, maintenance, and tinkering are not only material, everyday practices but also *discursive*. Reasoning with ‘tech love goggles’ and holding out for a ‘magic leap’ can be understood as a kind of discursive repair work, wherein the technological promises maintain their legitimacy even in the face of failure or mismatch.

Both tech love goggles and the magic leap reflect broader techno‐deterministic outlooks. They both reveal a tendency to absolve technology of blame and to attribute technological difficulties to human factors (cognitive decline, lack of user interest, etc.) and organisational constraints (insufficient staff, poor procurement, etc.). The kind of techno‐optimism that this reveals is not a naïve outlook on the role of technology in health and social care—there are many problems with technology (just rarely by fault of the technology itself). The care professionals that we interviewed did describe being faced with rather daunting systemic challenges, such as staffing shortages and time pressure. In the light of these challenges, we see it as understandable that care professionals cling to hopeful solutions—indeed, optimism may be a readily available and important coping strategy. However, as our analysis shows, this optimism is channelled in ways that align with—and inadvertently perpetuate—idealised techno‐futures, often overshadowing the messy present.

Lastly, we wish to highlight some of the implications that this might have for care in health and social care settings. A serious implication of reasoning with the magic leap and tech love goggles is that we risk discounting the present. Thinking with Mol (2008), we can see how the techno‐determinism and the techno‐optimism that inform ‘tech love goggles’ and ‘the magic leap’ fit squarely with a *logic of choice* rather than a *logic of care*. The logic of choice denotes a logic upon which most organised care is built; it assumes a rational, informed, and autonomous care recipient in a stable, standardised, and coherent institutional setting. Mol argues that this logic of choice is frequently at odds with a logic of care. Within the logic of care, care is oriented towards the ongoing work of responding to bodies, needs and situations as they unfold in practice, rather than towards the predefined choices or the anticipation of future solutions. Care is thus understood as situated, adaptive, and inherently uncertain, requiring continuous adjustment, tinkering and attentiveness to what is possible here and now, rather than faith in seamless trajectories from means to ends. Technologies, specifically, are treated as ‘unruly’ and ‘inventive mediators’ in Mol's framework (2008, 50). Care, from this perspective, involves a persistent attentiveness to how technologies behave in practice and a willingness to continuously recalibrate relations between people, tools and situations. Reasoning with ‘tech love goggles’ runs counter to this orientation as it tends to stabilise technology as essentially sound, locating problems in users, organisations or other contextual factors. The work of mutual adjustment that Mol identifies as crucial to good care is deferred or minimised, replaced by confidence that technologies will eventually align with practice once external obstacles are resolved. Within the logic of choice the connection between means and ends is simplified, much like it is in the ‘magic leap’. Seen from this perspective, postponing problems to a future technological resolution risks obscuring the ethical and practical labour involved in sustaining care in the present. This tension between future‐oriented techno‐promises and present‐oriented care practices provides a critical entry point for examining whose needs are prioritised, whose are deferred and with what consequences.

As a closing statement, we argue that discounting the present through tech love goggles and the magic leap has particularly serious ramifications for those under the care of our participants. When the present is discounted, and the future is mythologised, what becomes of those whose futures are, by definition, the most limited? For those working with the oldest of the old, the notion of a deferred digital utopia becomes not just inadequate but inappropriate. Our findings suggest that we need to take seriously not only the visions of the future being pursued, but also the realities of the present that are being neglected. Without this, we risk leaving frontline workers to manage broken promises with diminishing resources and expanding responsibilities, while continuing gazing at technology through tech love goggles, trusting in a magic leap.

## Author Contributions

**Freja Morris:** conceptualization, data curation, writing – original draft, writing – review and editing. **Fredrika Thelandersson:** investigation, data curation, methodology, project administration. **Helena Sandberg:** conceptualization, methodology, funding acquisition, supervision, writing – review and editing.

## Ethics Statement

This study was approved by the Swedish Ethical Review Authority (Etikprövningsmyndigheten; approval no. 2022‐05780‐01) on December 01, 2022.

## Consent

The authors have nothing to report.

## Conflicts of Interest

The authors declare no conflicts of interest.

## Permission to Reproduce Material From Other Sources

The authors have nothing to report.

## Data Availability

Research data are not shared.
